# Effect of the Premalignant and Tumor Microenvironment on Immune Cell Cytokine Production in Head and Neck Cancer

**DOI:** 10.3390/cancers6020756

**Published:** 2014-04-02

**Authors:** Sara D. Johnson, Anna-Maria A. De Costa, M. Rita I. Young

**Affiliations:** 1 Department of Microbiology and Immunology, Medical University of South Carolina, 173 Ashley Avenue, Charleston, SC 29425, USA; E-Mail: johsad@musc.edu; 2 Department of Otolaryngology, Head and Neck Surgery, Medical University of South Carolina, 135 Rutledge Avenue, Charleston, SC 29425, USA; E-Mail: clarkann@musc.edu; 3 Department of Medicine, Medical University of South Carolina, 96 Jonathan Lucas Street, Charleston, SC 29425, USA; 4 Medical Research Service (151), Ralph H. Johnson Veterans Affairs Medical Center, 109 Bee Street, Charleston, SC 29401, USA

**Keywords:** head and neck cancer, immunosuppression, tumor microenvironment, cytokines

## Abstract

Head and neck squamous cell carcinoma (HNSCC) is marked by immunosuppression, a state in which the established tumor escapes immune attack. However, the impact of the premalignant and tumor microenvironments on immune reactivity has yet to be elucidated. The purpose of this study was to determine how soluble mediators from cells established from carcinogen-induced oral premalignant lesions and HNSCC modulate immune cell cytokine production. It was found that premalignant cells secrete significantly increased levels of G-CSF, RANTES, MCP-1, and PGE_2_ compared to HNSCC cells. Splenocytes incubated with premalignant supernatant secreted significantly increased levels of Th1-, Th2-, and Th17-associated cytokines compared to splenocytes incubated with HNSCC supernatant. These studies demonstrate that whereas the premalignant microenvironment elicits proinflammatory cytokine production, the tumor microenvironment is significantly less immune stimulatory and may contribute to immunosuppression in established HNSCC.

## 1. Introduction

Head and neck squamous cell carcinoma (HNSCC) accounts for over 90% of all head and neck cancers, with approximately 650,000 cases diagnosed each year worldwide [1,2]. Because of the aggressive nature of the disease, the five-year survival rate remains around 50%, despite some advances in treatment over that last 30 years. HNSCC patients are characterized by systemic immunosuppression, exhibiting increased populations of regulatory T cells and CD34^+^ progenitor cells, which suppress CD8^+^ T cell- and CD4^+^ helper T cell-mediated immunity at the primary tumor site and are associated with a poorer prognosis [3,4,5,6]. It is well defined that established HNSCC is associated with increased levels of suppressive immune cells [6,7], but how the tumor directly modulates the immune response is less clear. 

A significant complication of understanding how the tumor contributes to immunosuppression is the complex network of tumor cells, fibroblasts, carcinoma-associated fibroblasts (CAFs), smooth muscle cells, endothelial cells, and tumor-infiltrating immune populations including tumor-associated macrophages, B cells, T cells and antigen-presenting cells that make up the tumor microenvironment [8,9,10]. Some of the mechanisms by which HNSCC tumors evade host immune recognition, including down-regulating MHC class I/II and costimulatory molecules and upregulating FasL and PD-L1, have been defined [11,12,13,14,15]. One of the major mechanisms by which HNSCC tumors are thought to evade host immune recognition is by modulating the cytokine environment at the tumor site. By secreting cytokines such as IL-6 and IL-10, HNSCC tumor cells promote a Th2-skewed response, which is associated with decreased antitumor efficacy [10,16]. This Th2-skewing is also evident systemically, as PBMCs isolated from advanced HNSCC patients secrete abnormally high levels of Th2 cytokines [17,18]. Along with Th2-skewed cytokines, HNSCC tumors secrete increased levels of immunosuppressive factors such as TGF-β that function to directly inhibit cytotoxic T cell-mediated immunity and recruit immunosuppressive cells, including myeloid-derived suppressor cells (MDSCs) and M2-skewed macrophages, to the tumor site [19,20]. Once at the tumor site, HNSCC tumor cells harness these immunosuppressive cells for several tumor-promoting functions, including increased growth and angiogenesis. HNSCC cells trigger increased IL-6 production from CD34^+^ progenitor cells, for example, promoting angiogenesis in the tumor microenvironment [21]. HNSCC tumors also secrete factors that are typically associated with a proinflammatory response, harnessing these immune modulators to favor growth, angiogenesis and paradoxically, immune escape. GM-CSF and PGE_2_, factors that have traditional proinflammatory roles that support the differentiation of macrophages and neutrophils in the early stages of inflammation, are produced by the HNSCC tumors to favor growth, angiogenesis, and paradoxically, immune escape. [22]. Tumor-secreted GM-CSF has been shown to promote MDSC recruitment and differentiation, and high levels of GM-CSF in HNSCC patients are associated with a poorer prognosis [5,23]. Increased levels of PGE_2_ are associated with invasion and angiogenesis in aggressive early-stage tumors [24]. Other factors secreted by HNSCC tumors, including monocyte chemotactic protein 1 (MCP-1), have been shown to contribute to immunosuppression at the tumor site by recruiting a population of IL-10 and TGF-β-secreting M2 skewed tumor-associated macrophages [25]. By secreting a host of immune modulators, HNSCC tumors thwart an effective immune response, presenting a significant challenge for treatment.

A critical gap that remains in head and neck cancer research is how the immune response evolves from premalignancy to the established tumor state. Studies using the 4-nitroquinoline 1-oxide (4-NQO) mouse model of oral carcinogenesis offer some insight into the immune changes that take place during the development of HNSCC. This model is based on the carcinogenic effects of 4-NQO, which mimic the effects of tobacco, a major risk factor for HNSCC [26,27]. Although HNSCC-bearing mice are characterized by an increase in conventional CD4^+^ and CD8^+^ T cells in tumor-draining lymph nodes compared to premalignant-bearing and control mice, these cells exhibit functional deficits and have significantly decreased proliferative capacity [28]. In contrast, the premalignant state is characterized by a significantly increased population of conventional T cells bearing markers for activation, memory, and exhaustion compared to HNSCC-bearing mice, indicating that an active immune response is taking place in the preneoplastic stage and that this response is thwarted once the tumor becomes established. In the 4-NQO mouse model, the premalignant state is also characterized by a significant increase in IL-17A-secreting Th17 cells in tumor-draining lymph nodes compared to HNSCC-bearing mice [28]. Taken together, these data suggest that while the premalignant environment supports a proinflammatory immune response, the established tumor state does not.

The focus of the present study is to determine how the premalignant microenvironment differs from the tumor microenvironment, and how this might be modulating the immune response during oral carcinogenesis. To address this question, premalignant lesion and HNSCC cell lines, respectively, were established from the 4-NQO mouse model of oral carcinogenesis and supernatant was collected for cytokine/chemokine/prostaglandin analysis. The effect of premalignant lesion cell and HNSCC cell supernatant on immune cell cytokine production was analyzed by culture with splenocytes harvested from control C57/BL6 mice and cytokine/chemokine analysis of collected supernatant. Splenocytes were chosen for this study because total cell numbers harvested from cervical lymph nodes of control C57/BL6 mice were not sufficient for measurement of detectable cytokine/chemokine levels. Based on previous studies using the 4-NQO model, it was hypothesized that premalignant lesion cells would secrete increased levels of proinflammatory mediators compared to HNSCC tumor cells, which would lead to an increased production of proinflammatory cytokines by splenocytes cultured with premalignant supernatant compared to HNSCC supernatant. The current study verifies that premalignant lesion cells secrete an increased level of several proinflammatory mediators, including G-CSF and PGE_2_, compared to HNSCC cells *in vitro*. When cultured with splenoctyes, premalignant lesion cell supernatant elicits a significant proinflammatory response, stimulating the production of Th1-, Th2-, and Th17-associated cytokines, whereas HNSCC supernatant is significantly less immune stimulatory.

## 2. Results and Discussion

### 2.1. Premalignant Lesion Cells Release Significantly Increased Levels of Pro-Inflammatory Mediators Compared to HNSCC Cells in Vitro

In HNSCC, the tumor microenvironment is shaped, in part, by the cytokine profile of the tumor cells themselves. Previous studies have shown that a panel of proinflammatory cytokines, including IL-1α, IL-6, and GM-CSF, are secreted from both murine and human SCC cell lines and freshly isolated primary HNSCC tumors [22,29,30]. The profile of proinflammatory mediators released by premalignant lesion cells—and how this compares to HNSCC cells—has not been extensively investigated. To address this question, premalignant lesion and HNSCC cell lines were established from the 4-NQO mouse model of oral carcinogenesis and supernatant was collected for cytokine/chemokine/prostaglandin analysis. 

**Figure 1:**
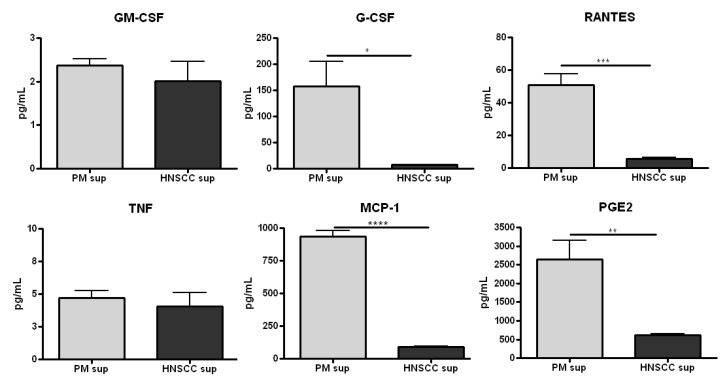
Premalignant lesion cells release significantly increased levels of pro-inflammatory mediators compared to HNSCC cells *in vitro*. Cells were grown to confluency and supernatant was collected after 48 h for cytokine/chemokine analysis. Data shown represent three independent experiments performed in duplicate. * *p* < 0.05 ** *p* < 0.01 *** *p* < 0.001 **** *p* < 0.0001.

Although the level of GM-CSF secreted from premalignant lesion cells and HNSCC cells was not significantly different (Figure 1), it was found that premalignant lesion cells release a significantly higher level of G-CSF than HNSCC cells (over 20-fold) *in vitro* (Figure 1). In addition, premalignant cells release a significantly increased level of the Th1-associated chemokine RANTES compared to HNSCC cells (10-fold) (Figure 1). Furthermore, the level of PGE_2_ secreted by premalignant cells, about 2,500 pg/mL, was over 4-fold the level secreted by HNSCC cells *in vitro* (Figure 1). The levels of other tumor-secreted cytokines/chemokines, including IL-1α, IL-6, IL-10, and TNF were negligible and/or did not significantly differ between premalignant and HNSCC supernatants (all data not shown). Taken together, these data suggest that the premalignant lesion microenvironment differs from the HNSCC microenvironment and that significant immune-modulating changes in the chemokine and prostaglandin environment are occurring earlier in the progression of HNSCC.

### 2.2. Splenocytes Cultured with Premalignant Supernatant Secrete Significantly Increased Levels of Innate Proinflammatory Mediators Compared to Splenocytes Cultured with HNSCC Supernatant

To investigate how factors produced by premalignant lesion and HNSCC tumor cells, respectively, might be modulating cytokine/chemokine release from immune cells, splenocytes were cultured with premalignant or HNSCC cell-conditioned media, and supernatant was collected for cytokine/chemokine analysis. Previous studies using the 4-NQO model have shown that cervical lymph node cells from both premalignant and HNSCC-bearing mice release an increased amount of Th1/Tc1-associated chemokines (RANTES, MIP-1α, MIP-1β) upon stimulation compared to control, suggesting that immune cells in premalignant lesion and tumor-draining lymph nodes are more activated/pro-inflammatory than the control state [28]. Because our preliminary studies have shown that premalignant lesion cells produce significantly increased levels of proinflammatory mediators compared to HNSCC cells, we hypothesized that premalignant supernatant would elicit increased production of proinflammatory chemokines compared to HNSCC supernatant. 

**Figure 2:**
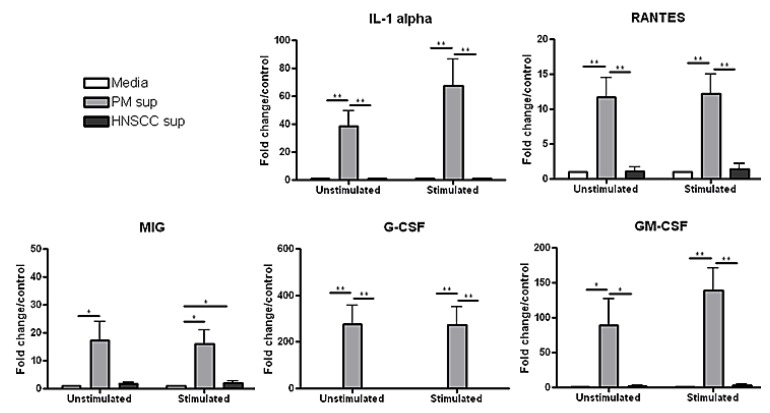
Splenocytes cultured with premalignant supernatant release significantly increased levels of innate proinflammatory mediators compared to splenocytes cultured with HNSCC supernatant or media alone. Splenocytes were incubated with media, premalignant cell-conditioned supernatant, or HNSCC cell-conditioned supernatant, respectively, and supernatant was collected for cytokine/chemokine analysis after 72 h. Data shown represent 3 independent experiments performed in duplicate. * *p* < 0.05 ** *p* < 0.01.

As shown in Figure 2, the levels of proinflammatory IL-1α and Th1-associated RANTES secreted by splenocytes in the presence of premalignant supernatant were significantly increased compared to levels secreted by spleen cells that were incubated with HNSCC supernatant or media alone, with and without stimulation with PMA/ionomycin. The level of IL-1α secreted by splenocytes in premalignant supernatant increased from about 40-fold/control to almost 70-fold/control after stimulation, whereas the level of RANTES secreted by splenocytes was about 12-fold/control with and without stimulation, respectively, *in vitro*. There were no significant differences in the levels of MIP-1α and MIP-1β secreted by splenocytes in the presence of premalignant and HNSCC supernatant, respectively, compared to control (data not shown).

In addition to stimulating increased production of IL-1α and RANTES, premalignant supernatant elicited increased production of the granulocyte/macrophage-associated chemokines MIG, G-CSF and GM-CSF from splenocytes compared to HNSCC supernatant or media alone, with and without stimulation. The amount of MIG secreted by splenocytes in the presence of premalignant supernatant was about 15-fold the level secreted by splenocytes in the presence of HNSCC supernatant or media alone, with and without stimulation. Similarly, the level of G-CSF secreted by splenocytes in the presence of premalignant supernatant was over 275-fold the level secreted by splenocytes in the presence of HNSCC supernatant or media alone. The level of GM-CSF secreted by splenocytes in the presence of premalignant supernatant was 88-fold/control, increasing to about 140-fold/control after stimulation *in vitro*, whereas the level secreted by splenocytes in the presence of HNSCC supernatant was 2-fold/control, increasing to only 3-fold/control after stimulation. 

These data suggest that while the premalignant microenvironment stimulates a significant proinflammatory innate response, the HNSCC microenvironment is significantly less immune stimulatory and may be a key mechanism by which the HNSCC tumor escapes an effective immune response.

### 2.3. Splenocytes Cultured with Premalignant Supernatant Secrete Significantly Increased Levels of Th1-, Th2-, and Th17-Associated Cytokines Compared to Splenocytes Cultured with HNSCC Supernatant

Previous studies have shown that factors in the HNSCC tumor microenvironment, including IL-6, IL-10 and PGE_2_, modulate the activity of infiltrating lymphocytes. Tumor-infiltrating T cells exhibit a number of functional defects, including decreased proliferation in response to IL-2, decreased expression of the CD3 zeta chain, and an imbalanced cytokine profile, marked by significantly decreased IL-2 and IFN-γ secretion, suggesting a less activated phenotype [31,32]. However, the effect of the tumor microenvironment on immune cell cytokine production is not so straightforward. Studies based on the 4-NQO model have shown that cervical lymph node cells from both premalignant and HNSCC-bearing mice secreted significantly increased levels of the Th1/Tc1-associated cytokines IL-2 and IFN-γ compared to control, suggesting that the tumor-draining lymph nodes of both premalignant and HNSCC mice promote inflammatory cytokine production [28]. To investigate how factors produced by premalignant lesion and HNSCC cells, respectively, might be altering cytokine production by immune cells/T cells, splenocytes were cultured with premalignant or HNSCC-conditioned media and supernatant was collected for cytokine analysis.

**Figure 3:**
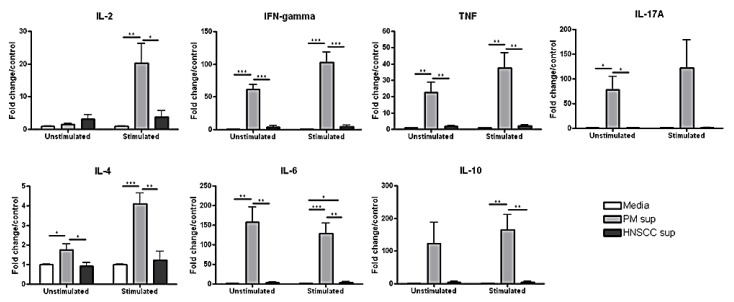
Splenocytes cultured with premalignant supernatant secrete significantly increased levels of Th1-, Th2-, and Th17-associated cytokines upon stimulation compared to splenocytes cultured with HNSCC supernatant or media alone. Splenocytes were incubated with media, premalignant cell-conditioned supernatant, or HNSCC cell-conditioned supernatant, respectively, and supernatant was collected for cytokine analysis after 72 h. Data shown represent 3 independent experiments performed in duplicate. * *p* < 0.05, ** *p* < 0.01, *** *p* < 0.001, **** *p* < 0.0001.

As shown in Figure 3 (below), we found that the level of IL-2 secreted by splenocytes cultured with premalignant supernatant was significantly higher (20-fold/control) than splenoctyes cultured with HNSCC supernatant (3-fold/control), after stimulation *in vitro.* Similarly, the levels of Th1-associated IFN-γ and TNF were significantly higher (over 60-fold/control and 20-fold/control, respectively) in the supernatant of splenocytes cultured with premalignant lesion cell supernatant compared to HNSCC cell supernatant (about 4-fold/control and 2-fold/control, respectively), without stimulation. The levels of IFN-γ and TNF increased to over 100-fold/control and 37-fold/control, respectively, after stimulation in the presence of premalignant supernatant. This suggests that the premalignant microenvironment supports a Th1-type response, whereas the tumor microenvironment does not.

Previous studies using the 4-NQO mouse model of oral carcinogenesis have shown that the percentage and absolute number of IL-17A-secreting Th17 cells are significantly increased in the tumor-draining lymph nodes of premalignant mice compared to HNSCC-bearing and control mice [28]. To investigate how the premalignant and HNSCC microenvironment might be modulating IL-17A secretion from tumor-infiltrating immune cells, control splenocytes were cultured with premalignant or HNSCC supernatant, respectively, and supernatant was collected for cytokine analysis. Splenocytes cultured with premalignant supernatant secreted significantly higher levels of IL-17A (78-fold/control) than splenocytes cultured with HNSCC supernatant (1.2-fold/control), even without re-stimulation. After stimulation with PMA/ionomycin, the amount of IL-17A secreted by splenocytes in the presence of premalignant supernatant increased to over 120-fold/control, whereas the amount of IL-17A released in the presence of HNSCC supernatant increased only slightly to 1.4-fold/control. This suggests that while the premalignant microenvironment elicits a significant Th17-type response, the HNSCC microenvironment does not.

Although previous studies have shown that the tumor microenvironment is Th2-skewed, we found that HNSCC supernatant does not directly elicit a significant Th2-type response *in vitro*. The levels of Th2-associated IL-4 and IL-6 secreted by splenocytes cultured with premalignant lesion cell supernatant were significantly higher than splenocytes cultured with HNSCC cell supernatant or media alone. Before stimulation, the levels of IL-4 and IL-6 secreted by splenocytes in the presence of premalignant supernatant were approximately 2-fold/control and over 150-fold/control, respectively. After stimulation *in vitro*, these levels rose to over 4-fold/control for IL-4 and decreased slightly to 130-fold/control for IL-6. The level of IL-10, also associated with a Th2-type immune response, was significantly increased in the supernatant of splenocytes cultured with premalignant lesion cell supernatant (about 165-fold/control) compared to HNSCC cell supernatant (4-fold/control), after stimulation. Overall, these data suggest that premalignant lesion supernatant elicits a significant Th1-, Th2-, and Th17-associated response, whereas HNSCC supernatant is significantly less immune stimulatory.

## 3. Experimental

### 3.1. Oral HNSCC Carcinogenesis

Five mg/mL 4-NQO in propylene glycol stock was administered in the drinking water (at a final concentration of 50 μg/mL) of 2-month-old female C57BL/6 mice (Charles Rivers Laboratory, Wilmington, MA, USA) until the development of premalignant oral lesions (6–8 weeks) or HNSCC (12–16 weeks). To monitor lesion development, mice were endoscoped weekly using a Stryker 1.9 mm × 30 scope and a Stryker 1088 camera (Stryker, Kalamazoo, MI, USA). During the procedure, mice were sedated with inhaled isoflurane (Piramal Healthcare, Bethlehem, PA, USA). 

### 3.2. Premalignant Lesion and HNSCC Cell Lines

Primary cell lines were established by excising the tongues of premalignant or HNSCC-bearing mice at the appropriate stage, as defined through histopathological analysis by the oral pathology section in the Center for Oral Health Research at the Medical University of South Carolina. Premalignant lesion cells or HNSCC carcinoma cells, respectively, were extracted from the tongues and cultured in 75 cm^2^ flasks in DMEM media supplemented with 10% FBS and penicillin/streptomycin/amphotericin B at 37 °C. To establish cell lines, premalignant lesion cells or HNSCC tumor cells, respectively, were washed several times with PBS over 72 h. Remaining lesion or carcinoma cells, respectively, were removed and transferred to a fresh 75 cm^2^ flask in 10% DMEM media for culture at 37 °C for several weeks. After cell lines were established, supernatant was collected after 48 h of culture (when cells reached confluence) for cytokine analysis. Prior to defining cells as premalignant or HNSCC, their epithelial phenotype was confirmed as well as uniformity of their microscopic and growth characteristics.

### 3.3. Spleen Processing

Spleens were harvested from control C57BL/6 mice and homogenized using a glass homogenizer. Cells were passed through a 40-μm cell strainer (BD Falcon, San Jose, CA, USA) and rinsed with Hank’s Buffered Saline Solution (HBSS, Invitrogen, Grand Island, NY, USA). Red blood cells were lysed by adding ACK Lysing Buffer (BioWhittaker, Walkersville, MD, USA) for 3 min. Splenocytes were then washed twice with HBSS. Cell number was determined by counting cells excluding trypan blue using a hemocytometer. 

### 3.4. Splenocyte Cultures

Each time an experiment was performed, it used premalignant cell or tumor cell supernatant derived from a single premalignant-bearing or HNSCC tumor-bearing mouse, respectively. The experiments were then repeated. The supernatants were cultured with splenocytes from a total of three control C57BL/6 mice and cytokine release was analyzed as a measure of fold change/control for each individual control mouse, in duplicate. Control splenocytes were plated 1 × 10^6^ cells/well in a 12-well tissue plate pre-coated with anti-mouse CD3ε with 30IU recombinant mouse IL-2 (R&D Systems, Minneapolis, MN, USA) in 10% DMEM media, premalignant lesion cell-conditioned media, or HNSCC cell-conditioned media for 72 h at 37 °C. Premalignant lesion cell-conditioned media and HNSCC cell-conditioned media, respectively, were diluted 1:2 in 10% DMEM media to culture with splenocytes. Cells were then restimulated for 4 h with 50 ng/mL PMA and 1 μg/mL ionomycin. Supernatant was collected for cytokine analysis. For each of three independent experiments, splenocytes from a control C57/BL6 mouse were cultured with media, premalignant lesion cell-conditioned or HNSCC cell-conditioned media, respectively. 

### 3.5. Cytokine Bead Array

All reagents used for the cytokine bead array were from BD Biosciences unless otherwise specified. The levels of IFN-γ, IL-2, IL-17A, IL-4, IL-6 and IL-10 in cell supernatant were determined using a mouse cytometric bead array Th1/Th1/Th17 cytokine kit. Levels of IL-1α, IL-1β, IL-13, IL-12p70, IL-9, GM‑CSF, G-CSF, MIG, MCP-1, MIP-1α, MIP-1β, and RANTES in cell supernatant were determined using cytometric bead array flex sets for the individual cytokines according to the manufacturer’s instructions. A FACS Canto (Becton Dickinson, San Jose, CA, USA) flow cytometer was used to quantify cytokine profiles and relative amounts of each cytokine were analyzed using FCAP Array Software (manufactured by Soft Flow Hungary Ltd. for BD Biosciences, St. Louis Park, MN, USA). Cytokine levels in total splenocyte cell supernatant were adjusted for baseline levels in premalignant or HNSCC cell supernatant, respectively, for analysis. To analyze fold change/control, cytokine levels in the supernatant of splenocytes cultured with premalignant or HNSCC cell-conditioned media, respectively, were compared to cytokine levels in the supernatant of splenocytes cultured with media alone for each of three independent experiments. 

### 3.6. Prostaglandin E_2_ ELISA

To quantify PGE_2_ in cell culture supernatant, a competitive ELISA was performed according to the manufacturer’s instructions (Thermo Scientific, Rockford, IL, USA). Premalignant and HNSCC cell supernatant was diluted 1:2 or 1:4 for analysis. Samples were tested in duplicate for each of three independent experiments. Optical density at 405 nm was analyzed using a Spectra Max M2 plate reader. Concentration of PGE_2_ was determined using SoftMax Pro 5.4.2 plate reader software (Molecular Devices, Sunnyvale, CA, USA). A standard concentration curve was generated for each independent experiment. 

### 3.7. Statistical Analysis [33]

A one-way ANOVA analysis was initially performed to determine if there were any significant differences in cytokine/chemokine production by splenocytes between control, premalignant lesion supernatant and HNSCC supernatant conditions. If differences were identified by the ANOVA analysis, a student’s *t* test was then performed to determine significance of differences between each of two groups (ex.: control *vs.* premalignant, control *vs.* HNSCC, premalignant *vs.* HNSCC). Data were reported using the mean as a measure of central tendency ± standard error of the mean. Significance was reported in the 95% confidence interval.

## 4. Conclusions

Whereas premalignant lesion cells from the 4-NQO mouse model of oral carcinogenesis release a panel of proinflammatory mediators including G-CSF, RANTES, MCP-1, and PGE_2_, HNSCC cells are characterized by significantly decreased secretion of these mediators. Furthermore, splenocytes cultured with premalignant lesion cell supernatant secrete significantly increased levels of Th1-, Th2-, and Th17-associated cytokines and chemokines compared to splenocytes cultured with HNSCC supernatant. This suggests that the premalignant microenvironment is significantly more immune stimulatory than the microenvironment of the established tumor in HNSCC, though the anti-tumor *vs.* pro-carcinogenic role of this response is obscure.

Previous studies have shown that HNSCC tumors modulate the cytokine environment to escape an effective immune response and enhance growth and metastasis. Tumor-secreted GM-CSF is involved in recruiting a population of MDSCs, which have been shown to inhibit IL-2 production from anti-CD3-activated intratumoral T cells and interfere with CD8^+^ T cell-mediated immunity at the tumor site [23,34,35]. Although high levels of GM-CSF have been detected in several human SCC lines, we did not find a significant level of GM-CSF in HNSCC supernatant (or premalignant supernatant). However, splenocytes cultured with premalignant supernatant secreted a significantly higher level of GM-CSF compared to splenocytes cultured with HNSCC supernatant or media alone, suggesting that factors in the premalignant environment elicit GM-CSF secretion from infiltrating immune cells. This may be a mechanism by which developing premalignant lesions recruit MDSCs. 

Another hematopoietic cytokine secreted by HNSCC tumor cells, G-CSF, has been shown to increase the invasiveness of tumors via upregulation of MMP-2 and is associated with a worse prognosis in patients [36,37,38]. In our study, the level of G-CSF secreted by premalignant lesion cells was significantly higher than HNSCC cells and splenocytes cultured with premalignant supernatant secreted significantly more G-CSF than splenocytes cultured with HNSCC supernatant. A similar trend was found with MCP-1, a chemokine that has previously been shown to recruit IL-1- and TNF-α-secreting macrophages to sustain VEGF secretion by tumor cells in a proangiogenic signaling loop [25]. Levels of MCP-1 were significantly increased in premalignant supernatant compared to HNSCC supernatant. RANTES, another proinflammatory chemokine that has been implicated in promoting HNSCC progression, was also significantly upregulated in premalignant supernatant compared to HNSCC supernatant [39]. These data suggest that the upregulation of several protumoral mediators may be taking place earlier in the carcinogenesis of HNSCC than has previously been appreciated. 

Although is well established that arachadonic acid metabolism is altered in established HNSCC tumors, the role of PGE_2_ in HNSCC carcinogenesis is not clearly defined. COX-2, the enzyme responsible for PGE_2_ production, is significantly upregulated in HNSCC tumors and increased levels of PGE_2_ are associated with decreased levels of CD8^+^ T cells and increased levels of suppressor cells at the tumor site [10,16,40,41]. PGE_2_ has also been shown to induce the production of IL-10 and directly suppress the production of proinflammatory cytokines by CD4^+^ T cells, implicating it as a key player in tumor-associated immunosuppression [39,42,43,44]. In our study, the level of PGE_2_ secreted by premalignant lesion cells was over four-fold the level secreted by HNSCC cells, suggesting that PGE_2_ synthesis might be upregulated earlier in the carcinogenesis of HNSCC, possibly as a mechanism to recruit suppressor cells and dampen the inflammatory response as the tumor becomes established. Future studies looking into the effect of COX-2 inhibitors on premalignant lesion cells and how this modulates immune cell cytokine production will help define the role of PGE_2_ in the progression of HNSCC.

Our studies suggest that the premalignant microenvironment is significantly more immune stimulatory than the HNSCC microenvironment. Splenocytes cultured with premalignant supernatant secrete significantly increased levels of the Th1-associated cytokines IL-2, IFN-γ, and TNF, Th17-associated IL-17A, and other proinflammatory mediators including RANTES and IL-1α compared to splenocytes cultured with HNSCC supernatant. Interestingly, premalignant supernatant also elicits increased secretion of Th2-associated IL-4, IL-6, and IL-10 from splenocytes compared to HNSCC supernatant. This is puzzling because IL-4 and IL-10 are known to play immunosuppressive roles and yet are being produced alongside proinflammatory cytokines in the presence of premalignant (but not tumor) supernatant. One explanation is that immune cells initially recognize dysplastic tissue and mount an inflammatory response at the premalignant stage, but factors released from the developing lesion, including G-CSF and PGE_2_, simultaneously recruit a population of immunosuppressive cells, which secrete factors such as IL-10 and TGF-β to dampen an effective Th1-mediated response as the tumor is established. At this point, the tumor has escaped immune surveillance and no longer needs to maintain as strong of a Th2-skewed environment to combat the resident inflammatory cells, which are significantly less active. To elucidate the mechanism by which factors produced by premalignant lesion cells and HNSCC cells, respectively, are modulating immune cell cytokine production, future studies will focus on the effect of lesion and tumor supernatant on isolated CD4^+^ and CD8^+^ T cells, F4/80^+^ macrophages, and CD11b^+^/CD11c^+^ dendritic cells.

These studies are not without limitation. First, these studies conducted analyses of tissue that had already developed into premalignant oral lesions or HNSCC. Future studies are also needed to determine the immunological impact of the early developmental stages of premalignant lesions prior to the visible appearance of the lesions. In addition, although established cell lines offer some insight into the cytokine environment of the premalignant lesion and HNSCC tumor, respectively, the degree to which they mimic the *in vivo* state is unknown. Transformation of the cells in culture may lead to the production of a different panel of cytokines/chemokines, which may change the effect of this supernatant on splenocyte cytokine production *in vitro*. Furthermore, these experiments do not take into account the myriad of immune mediators being produced by tumor-associated fibroblasts, endothelial cells, etc. in the tumor microenvironment and the interaction of tumor cells with these cells *in vivo.* However, because much is unknown about the premalignant state of HNSCC, analyzing the cytokine/chemokine profile of isolated premalignant lesion cells is an important first step to understanding how these cells might be modulating the immune response during HNSCC carcinogenesis.

HNSCC tumors use a variety of mechanisms to evade immune detection and thwart an effective host response, creating a significant obstacle for treatment in HNSCC patients. However, the mechanisms by which premalignant lesions modulate the immune response are largely unknown. This study offers insight into how the cytokine environment changes from premalignancy to established HNSCC and suggests that the first steps of immune escape may be taking place earlier in HNSCC carcinogenesis than has previously been appreciated, opening the door for more effective immunotherapy development.

## References

[1] Duray A., Demoulin S., Hubert P., Delvenne P., Saussez S. (2010). Immune suppression in head and neck cancers: A review. Clin. Dev. Immunol..

[2] Grandis J.R., Pietenpol J.A., Greenberger J.S., Pelroy R.A., Mohla S. (2004). Head and neck cancer: Meeting summary and research opportunities. Cancer Res..

[3] Bergmann C., Strauss L., Wang Y., Szczepanski M.J., Lang S., Johnson J.T., Whiteside T.L. (2008). T regulatory type I cells in squamous cell carcinoma of the head and neck: Mechanisms of suppression and expansion in advanced disease. Clin. Cancer Res..

[4] Badoual C., Hans S., Rodriguez J., Peyrard S., Klein C., Agueznay Nel H., Mosseri V., Laccourreye O., Bruneval P., Fridman W.H. (2006). Prognostic value of tumor-infiltrating CD4^+^ T-cell subpopulations in head and neck cancers. Clin. Cancer Res..

[5] Young M.R., Wright M.A., Lozano Y., Prechel M.M., Benefield J., Leonetti J.P., Collins S.L., Petruzzelli G.J. (1997). Increased recurrence and metastasis in patients whose primary head and neck squamous cell carcinomas secreted granulocyte-macrophage colony-stimulating factor and contained CD34^+^ natural suppressor cells. Int. J. Cancer.

[6] Chikamatsu K., Sakakura K., Whiteside T.L., Furuya N. (2007). Relationships between regulatory T cells and CD8^+^ effector populations in patients with squamous cell carcinoma of the head and neck. Head Neck.

[7] Drennan S., Stafford N.D., Greenman J., Green V.L. (2013). Increased frequency and suppressive activity of CD127(low/−) regulatory T cells in the peripheral circulation of patients with head and neck squamous cell carcinoma are associated with advanced stage and nodal involvement. Immunology.

[8] Koontongkaew S. (2013). The tumor microenvironment contribution to development, growth, invasion and metastasis of head and neck squamous cell carcinomas. J. Cancer.

[9] Badoual C., Sandoval F., Pere H., Hans S., Gey A., Merillon N., van Ryswick C., Quintin-Colonna F., Bruneval P., Brasnu D. (2010). Better understanding tumor-host interaction in head and neck cancer to improve the design and development of immunotherapeutic strategies. Head Neck.

[10] Young M.R. (2006). Protective mechanisms of head and neck squamous cell carcinomas from immune assault. Head Neck.

[11] Ferris R.L., Whiteside T.L., Ferrone S. (2006). Immune escape associated with functional defects in antigen-processing machinery in head and neck cancer. Clin. Cancer Res..

[12] Ogino T., Shigyo H., Ishii H., Katayama A., Miyokawa N., Harabuchi Y., Ferrone S. (2006). Hla class I antigen down-regulation in primary laryngeal squamous cell carcinoma lesions as a poor prognostic marker. Cancer Res..

[13] Grandis J.R., Falkner D.M., Melhem M.F., Gooding W.E., Drenning S.D., Morel P.A. (2000). Human leukocyte antigen class I allelic and haplotype loss in squamous cell carcinoma of the head and neck: Clinical and immunogenetic consequences. Clin. Cancer Res..

[14] Zeng X., Chen Q., Nie M. (2003). The relationship of Fas and FasL protein expression in oral carcinogenesis. West China J. Stomatol..

[15] Cho Y.A., Yoon H.J., Lee J.I., Hong S.P., Hong S.D. (2011). Relationship between the expressions of PD-L1 and tumor-infiltrating lymphocytes in oral squamous cell carcinoma. Oral Oncol..

[16] Jewett A., Head C., Cacalano N.A. (2006). Emerging mechanisms of immunosuppression in oral cancers. J. Dent. Res..

[17] Bose A., Chakraborty T., Chakraborty K., Pal S., Baral R. (2008). Dysregulation in immune functions is reflected in tumor cell cytotoxicity by peripheral blood mononuclear cells from head and neck squamous cell carcinoma patients. Cancer Immun..

[18] Lathers D.M., Achille N.J., Young M.R. (2003). Incomplete Th2 skewing of cytokines in plasma of patients with squamous cell carcinoma of the head and neck. Hum. Immunol..

[19] Lu S.L., Reh D., Li A.G., Woods J., Corless C.L., Kulesz-Martin M., Wang X.J. (2004). Overexpression of transforming growth factor beta1 in head and neck epithelia results in inflammation, angiogenesis, and epithelial hyperproliferation. Cancer Res..

[20] Wahl S.M., Wen J., Moutsopoulos N. (2006). TGF-beta: A mobile purveyor of immune privilege. Immunol. Rev..

[21] Nitsch S.M., Pries R., Wollenberg B. (2007). Head and neck cancer triggers increased IL-6 production of CD34^+^ stem cells from human cord blood. In Vivo.

[22] Chen Z., Malhotra P.S., Thomas G.R., Ondrey F.G., Duffey D.C., Smith C.W., Enamorado I., Yeh N.T., Kroog G.S., Rudy S. (1999). Expression of proinflammatory and proangiogenic cytokines in patients with head and neck cancer. Clin. Cancer Res..

[23] Bronte V., Chappell D.B., Apolloni E., Cabrelle A., Wang M., Hwu P., Restifo N.P. (1999). Unopposed production of granulocyte-macrophage colony-stimulating factor by tumors inhibits CD8^+^ T cell responses by dysregulating antigen-presenting cell maturation. J. Immunol..

[24] Hambek M., Baghi M., Wagenblast J., Schmitt J., Baumann H., Knecht R. (2007). Inverse correlation between serum PGE2 and T classification in head and neck cancer. Head Neck.

[25] Liss C., Fekete M.J., Hasina R., Lam C.D., Lingen M.W. (2001). Paracrine angiogenic loop between head-and-neck squamous-cell carcinomas and macrophages. Int. J. Cancer.

[26] Forastiere A., Koch W., Trotti A., Sidransky D. (2001). Head and neck cancer. N. Engl. J. Med..

[27] Schoop R.A., Noteborn M.H., Baatenburg de Jong R.J. (2009). A mouse model for oral squamous cell carcinoma. J. Mol. Histol..

[28] De Costa A.M., Schuyler C.A., Walker D.D., Young M.R. (2012). Characterization of the evolution of immune phenotype during the development and progression of squamous cell carcinoma of the head and neck. Cancer Immunol. Immunother..

[29] Smith C.W., Chen Z., Dong G., Loukinova E., Pegram M.Y., Nicholas-Figueroa L., van Waes C. (1998). The host environment promotes the development of primary and metastatic squamous cell carcinomas that constitutively express proinflammatory cytokines IL-1alpha, IL-6, GM-CSF, and KC. Clin. Exp. Metastasis.

[30] Mann E.A., Spiro J.D., Chen L.L., Kreutzer D.L. (1992). Cytokine expression by head and neck squamous cell carcinomas. Am. J. Surg..

[31] Whiteside T.L. (2005). Immunobiology of head and neck cancer. Cancer Metastasis Rev..

[32] Reichert T.E., Rabinowich H., Johnson J.T., Whiteside T.L. (1998). Mechanisms responsible for signaling and functional defects. J. Immunother..

[33] (2013). GraphPad Prism.

[34] Almand B., Clark J.I., Nikitina E., van Beynen J., English N.R., Knight S.C., Carbone D.P., Gabrilovich D.I. (2001). Increased production of immature myeloid cells in cancer patients: A mechanism of immunosuppression in cancer. J. Immunol..

[35] Pak A.S., Wright M.A., Matthews J.P., Collins S.L., Petruzzelli G.J., Young M.R. (1995). Mechanisms of immune suppression in patients with head and neck cancer: Presence of CD34^+^ cells which suppress immune functions within cancers that secrete granulocyte-macrophage colony-stimulating factor. Clin. Cancer Res..

[36] Ninck S., Reisser C., Dyckhoff G., Helmke B., Bauer H., Herold-Mende C. (2003). Expression profiles of angiogenic growth factors in squamous cell carcinomas of the head and neck. Int. J. Cancer.

[37] Sugimoto C., Fujieda S., Sunaga H., Noda I., Tanaka N., Kimura Y., Saito H., Matsukawa S. (2001). Granulocyte colony-stimulating factor (G-CSF)-mediated signaling regulates type IV collagenase activity in head and neck cancer cells. Int. J. Cancer.

[38] Tsuzuki H., Fujieda S., Sunaga H., Noda I., Saito H. (1998). Expression of granulocyte colony-stimulating factor receptor correlates with prognosis in oral and mesopharyngeal carcinoma. Cancer Res..

[39] Linkov F., Lisovich A., Yurkovetsky Z., Marrangoni A., Velikokhatnaya L., Nolen B., Winans M., Bigbee W., Siegfried J., Lokshin A. (2007). Early detection of head and neck cancer: Development of a novel screening tool using multiplexed immunobead-based biomarker profiling. Cancer Epidemiol. Biomark. Prev..

[40] Young M.R., Wright M.A., Lozano Y., Matthews J.P., Benefield J., Prechel M.M. (1996). Mechanisms of immune suppression in patients with head and neck cancer: Influence on the immune infiltrate of the cancer. Int. J. Cancer.

[41] Abrahao A.C., Castilho R.M., Squarize C.H., Molinolo A.A., dos Santos-Pinto D., Gutkind J.S. (2010). A role for Cox2-derived PGE2 and PGE2-receptor subtypes in head and neck squamous carcinoma cell proliferation. Oral Oncol..

[42] Bao Y.S., Zhang P., Xie R.J., Wang M., Wang Z.Y., Zhou Z., Zhai W.J., Feng S.Z., Han M.Z. (2011). The regulation of CD4^+^ T cell immune responses toward Th2 cell development by prostaglandin E2. Int. Immunopharmacol..

[43] Kalinski P. (2012). Regulation of immune responses by prostaglandin E2. J. Immunol..

[44] MacKenzie K.F., Clark K., Naqvi S., McGuire V.A., Noehren G., Kristariyanto Y., van den Bosch M., Mudaliar M., McCarthy P.C., Pattison M.J. (2013). PGE(2) induces macrophage IL-10 production and a regulatory-like phenotype via a protein kinase A-SIK-CRTC3 pathway. J. Immunol..

